# "The Hospital" Nursing Mirror

**Published:** 1901-02-16

**Authors:** 


### "The Hospital" Nursing Mirror.

Being the Nursing Section of "The Hospital."

[Contributions for this Section of "The Hospital" should be addressed to the Editor, "The Hospital" Nursing Mirror, 28 & 29 Southampton Street Strand, London, W.C.]

### Notes on News from the Nursing World.

### THE SISTER WHO NURSED THE HEIR APPARENT.

Miss Georgina Hallam, senior sister at St. Mary's Hospital, who was summoned to Osborne to nurse the Duke of Cornwall and York during his attack of German measles, has nursed his Royal Highness on a former occasion. In Victoria Ward, over Sister Victoria's table, hangs a large oil painting of the Duke given to her, and in her little sitting-room are photographs of the King and Queen, and of the Duke and Duchess of Cornwall and York, which were also presented to her. Sister Victoria was first sent for by the Princess of Wales in 1891 to nurse Prince George when he had enteric. She had not returned to her hospital duties for many weeks when she was again telegraphed for to nurse the late Duke of Clarence. Since that time Sister Victoria has been honoured by a visit in her own little sitting-room from the present King, when he laid the foundation-stone of the Clarence wing, and from the Duke of Cornwall and York. The latter has more than once visited the hospital as its president, and has always received a hearty welcome. Sister Victoria's stay at Osborne was not long. She returned to the hospital on Tuesday night, after a week's absence, but before she left she had the pleasure of knowing that her Royal patient was completely restored to health. Miss Hallam has spent all her nursing days at St. Mary's Hospital. She was trained there, and has for many years been in charge of the largest medical ward—in fact, of three wards, Victoria, Carlisle, and Boynton.

### NURSES IN A TRAIN ATTACKED BY BOERS.

An attack was made last week upon a train on the Natal line by 400 Boers, who fired among the passengers. Several of the latter were nurses, and a bullet went through the hat of one of them. This did not exhaust the cowardly conduct of the enemies, for not content with firing on civilians and women, so obviously entitled to protection, the Boers robbed one of the nurses of £25. We trust that she will receive compensation from the British Government for her loss.

### AN EASY CURE.

During the present war, a Boer was admitted to the ward of a small up-country hospital. He described himself as suffering from many different symptoms; but the nurse's opinion was that his chief complaint was want of cleanliness. He was made to submit, with evident distaste, to the usual preliminary bath, and next morning he was pronounced well, and able to leave the hospital.

### A TRIBUTE TO THE EFFICACY OF FEMALE NURSING.

Perhaps the most interesting feature to nurses in the letter in Wednesday's *Times* of Mr. Burdett-Coutts, M.P., on the South African Hospitals Commission Report is his reference to the nursing at the Volks Hospital, Bloemfontein. He says:—

The Volks Hospital at Bloemfontein was the only one in which the patients were properly nursed by female nurses. Its principal doctor was Dr. Kellner, formerly mayor of Bloemfontein; its matron, Miss Maud Young, a strong, able, and efficient superintendent, who had a staff of eight nurses under her. There was no R.A.M.C. doctor or trained orderly in the hospital. The equipment was plain, simple, convenient, but in no way elaborate or luxurious. As admitted by the report with regard to the private hospitals, the serious cases gravitated to this one also. Its proportion of admitted convalescents would be nil. It had to deal with the epidemic under the same conditions of pressure, climate, provisions, water, and other matters, as the rest of the hospitals around it; but, owing to the untiring energy and devotion of its matron and female staff, it was properly nursed. What was the result? The general enteric mortality in Bloemfontein was 21 per cent. The same mortality in the Volks Hospital was 7·75.

This is the best practical proof that could be given of the value of the female nursing of sick and wounded soldiers.

### HOW TO SUPPRESS SHAM NURSES.

With reference to the correspondence in a daily paper —which erroneously speaks of Sir Henry Burdett as an advocate of the State Registration of nurses—respecting bogus nursing homes, there is one easy and obvious method by which the friends of patients can ascertain whether the nurse sent by an institution is properly qualified for her duties. By consulting "Burdett's Official Nursing Directory," they can speedily learn her antecedents and training. One of the many objections to State Registration is undoubtedly the fact that a nurse who had once been placed on the register would remain on it, though she might not have pursued her calling for years, and was quite out of touch with the latest requirements. "Burdett's Official Nursing Directory," being published and carefully revised annually, is always up to date, and gives the details of each nurse's career down to the time of issue. We do not say that there are no fully-trained and capable nurses whose names do not appear in its pages; but it contains such a large proportion of those who merit this description that private individuals who require nurses would experience no inconvenience in refusing to receive any others. Moreover, if all nurses fully understood that the exclusion of their names from the Directory, which, in the case of those who are adequately trained is only due to their own carelessness, seriously interfered with their professional earnings, they would themselves hasten to make the work even more comprehensive than it already is.

### TWO CONCEPTIONS OF THE NURSING AVOCATION.

A correspondent of a Yorkshire paper is very angry with the patient at a London hotel who, in reply to the question of his newly arrived nurse "Where am I to have my meals?" answered, "With the housekeeper I should think," and also with us for suggesting that hotel managers are obliged to consider the customers who object to the appearance of nurses in uniform at *table d'hôte*. We observed at the time that the patient may have lacked tact, but we are not sure that we did him justice. The housekeeper at a large hotel is usually an educated lady, with whom a nurse, if she be also an educated lady, would be glad to have her meals, and in the case in question there was not the shadow of cause for resentment. But "A Yorkshirewoman" is highly indignant at the idea of a nurse having her meals alone in her room. "Surely," she says, "that would not be a very enjoyable repast, nor should I think a nurse who would thus isolate herself would be a very cheerful or desirable companion in sickness." It is evident that "A Yorkshirewoman" quite misses the point. We share her desire that the lot of nurses, which is often very hard, should be made as cheerful as possible, but we repeat that the nurse who is so concerned about her own meals that she refuses to remain with a patient who does not meet her views by securing her a place at *table d'hôte*, displays the temper which is absolutely inconsistent with the avocation which she pursues. In striking contrast to the view which "A Yorkshirewoman" takes of nursing, is that of the rector of Christ Church, Kingstown, who, preaching at a special service for the nurses of the Dublin hospitals, said he wished them to ask themselves what was their chief aim as nurses?

Was it to have an honoured position; was it to make a living? They were honourable enough purposes in themselves; but, could they go further and say the chief aim of their life had something that would last for ever? If a woman had a low or a self-seeking purpose in life she might do very well for herself, but she had no right to take on any holy office; she had no right to be a nurse with the sacred ministry of the sick committed to her.

We should be sorry to see nurses making a parade of piety, but the nobler rather than the lower conception of the office of nurse is surely the one that ought always to be kept in mind.

### DEATH OF AN OLD GUY'S SISTER.

The death of Miss Barker, who was on the nursing staff of Guy's Hospital for a number of years, at the age of 75, has a pathetic interest. During her illness, arising from cancer of the throat, Miss Barker constantly dreamt that she was back at Guy's, where she spent nearly thirty years of her life. Entering the hospital in 1860, she was successively sister in Mary, Philip, Clinical, and Samaritan Wards. In 1881 she became housekeeper, and continued in that capacity until her retirement in 1889. Her quiet and unassuming manner, as well as her kind disposition and her devotion to her work, caused her to be greatly esteemed by the nurses under her; and so unobtrusive was her piety that only her intimate friends know that she worshipped at a Nonconformist chapel. Miss Barker received a pension for long service from the Governors of Guy's when she retired.

### THE ST. PATRICK'S NURSES' HOME.

The annual meeting of the friends and subscribers of the St. Patrick's Nurses' Home was held in Dublin on Friday, the Archbishop of Dublin in the chair. Countess Cadogan was among the distinguished company present. The Lord Mayor moved the adoption of the report, and we observe that the number of patients nursed during the year was 1,845, while the number of visits paid by nurses was 36,167. The balance-sheet showed a balance of £65 10s. 9d. to credit. Several excellent speeches were made at the meeting, notably that of Mr. Justice Fitzgibbon, who intimated his conviction that the work of the nurses in the slums of Dublin was harder than that of nursing sick and wounded soldiers in South Africa. Having also expressed his opinion that the women who made the choice of nursing as a profession selected the most burdensome occupation that a woman could adopt, he paid a warm tribute to the managers of St. Patrick's Home, as well as to the nursing staff, and pleaded that the latter should not be overworked, pointing out that the only way to prevent it was for the public to subscribe more money to provide more nurses.

### CRISIS AT WARE INFIRMARY.

The two nurses at the Workhouse Infirmary, Ware, have been informed in writing by the Guardians that they have decided to terminate their engagements at the end of a month, or the nurses may, if they prefer, send in their resignations. No reason whatever is given for this action, but some facts have come to our knowledge which perhaps throw light on the proceeding, if they do not altogether account for it. The infirmary contains less than 50 beds, nearly all of which are occupied. There are two nurses—one on duty during the day and the other at night. There are occasional lying-in patients, but most of the inmates in the sick wards are aged infirm chronic cases. Lunatics, we are informed, are admitted into the sick wards, and the nurse on duty gets no help, except in the day from the pauper ward. The male and female wards are separated by two large uncovered courtyards, and there is obviously nothing to prevent accidents occurring in one ward while the nurse is in the other. Again, when the Local Government Board Inspector last visited the little Hertfordshire town, he advised the nurses to ask the Guardians for whatever was necessary for the comfort of the patients. Three weeks ago, we understand, the Guardians were accordingly asked for spring beds, bed-cradles, and hot-water bottles. The existing beds are ticks of straw, hard and narrow. Five hot-water bottles have been sent in, but, instead of receiving the spring beds and bed-cradles, the nurses appear to have received notice to quit. They may easily go farther and fare better, if, as we are informed, there are no rules of any kind; no off-duty time, no bath, no comforts, and no sitting-room except their bedrooms, save part of a ward partitioned off and separated from the water sinks and closets by a folding door. The Guardians may depend upon it that, if these conditions are allowed to continue, capable nurses will not refrain from making humane requests on behalf of their patients, and changes will be constant at Ware.

### "NOT GUILTY; BUT DON'T DO IT AGAIN."

A case of special interest to nurses has been tried before the Lord Chief Justice of Ireland in the Nisi Prius Court at Dublin. The plaintiff was Miss Clare Gargan, a nurse at St. Vincent's Hospital, who suffered considerable injury from a collision between an outside car, on which she was being driven to duty, and an electric tram. There was no question as to the effects of the collision upon Miss Gargan, and the medical witnesses called on her behalf were not even cross-examined. But the Dublin United Tramways Company disputed their liability, and the Lord Chief Justice in charging the jury practically directed them not to give a verdict for the plaintiff. Having said that she had made a gallant effort, he contined:—"She was young and very attractive; but that was no reason why she should get a verdict if she had not a particle of a case. It would be a melancholy thing if the idea should go abroad that the electric tram was a sort of thing that every person on the street who met with an accident should have a cockshot at. If the Tramways Company were guilty of negligence no one would be more emphatic against them than he would be." In the face of this the jury could hardly, we suppose, do otherwise than acquit the defendants of being guilty of negligence; yet, curiously enough, they added "that the lady was entitled to receive something from the company." On what ground? Because, as the judge says, "She is young and very attractive?" However, we hope that she has got "something" from the board of directors. If half a loaf is better than no bread, voluntary compensation is preferable to none.

### AN INQUIRY AT NEWTON ABBOT INFIRMARY.

The nurse at Newton Abbot Workhouse Infirmary, to whom allusion was made last week, has resigned, and letters of resignation have been sent to the guardians by their other nurses. At a meeting of the Board of Guardians it was decided, after an animated discussion, to inquire into the cause of the resignations. Mr. Burridge protested against the cowardly and unmanly way in which Miss Flaherty—there seems no longer any reason for suppressing names—had been treated, and said "there was no charge against her, and nothing that she could answer." This is exactly the view we expressed in commenting upon "the mystery at Newton Abbot," and we can only hope that light will be thrown upon the matter in the course of the investigation which has been ordered, and pending which all the nurses have withheld their resignations. It is possible that the guardians may be able to justify the semi-secrecy they have observed, or that, as one of them alleges, they acted in "the interests of the nurse." But it seems to us that the interests of all the nurses, not to mention those of the ratepayers, demand that the fullest publicity should be given to the facts.

### MORE NURSES WANTED AT LEEDS.

In view of the many nurses ready to accept engagements, it is a curious feature of the twenty-fifth annual report of the Leeds Trained Nurses' Institution that, while the number of cases attended during the last twelve months was 967, no less than 391 had to be declined in consequence of no nurse being at liberty when application was made. One wonders why the staff is not increased. Yet the Institute has attached to it 96 nurses who are engaged in private nursing, and 13 probationers in training at hospitals. The superintendent has held her post ever since the Institution was founded, and conducts its affairs in the most admirable manner. A large portion of the balance of profits for the year had, it was stated at the meeting of members, been appropriated between the Leeds Trained Nurses' Institution Trust Fund of the Royal National Pension Fund for Nurses, and the Leeds District Nursing Institution. The latter continues to extend the scope of its operations, and the number of cases attended in 1900 was 2,694, nearly 60,000 visits being paid.

### THE RESIGNATIONS AT PORTSMOUTH INFIRMARY.

It is significant as to the feeling of the nurses attached to the Portsmouth Workhouse Infirmary that a farewell supper was given the other evening by three-and-twenty of the staff to the matron and assistant matron. The company assembled at 9.30 p.m., after duty hours, and speeches were made testifying to the kindness and consideration always shown by Miss Court and her colleague to their subordinates. Deep regret was expressed at the prospect of their departure, and not a discordant note was struck during the evening. After songs and recitations, "Auld Lang Syne" was given at midnight.

### AN OPENING FOR ASYLUM NURSES.

Among the Institutions of Cape Town, a correspondent mentions the Dalkenburgh Asylum. It is a very fine building, standing in its own grounds, and there are constantly vacancies on the staff for nurses who are competent to "minister to the mind diseased." They are paid at the rate of 2s. 3d. per diem, with laundry, uniform, and rations.

### THE NURSES' TEA-PARTY.

On Thursday last the district nurses of Harrington gave their annual Christmas tree to children who had themselves been patients and children of patients (preferably the poorest). A good tea, followed by a magic lantern, games, and some impromptu songs and recitations by the children themselves, all went to make up a very happy evening for the ninety guests assembled. The crowning point was the distribution of presents, each child receiving a useful garment or a toy, also an orange and sweets, after which they were allowed to go home, all eager to tell of the delights of "The Nurses' Tea-party."

### SHORT ITEMS.

The Earl of Dysart has offered £500 towards a sum of £3,000 required to erect a nurses' institute and laundry in connection with the Liverpool Hahnemann Hospital, on condition that the balance of the money be raised before the 4th of next August.—Lady Curzon has issued a personal appeal for funds to form a Victoria scholarship in connection with the Dufferin funds for training native midwives to work solely in zenanas in the outlying districts.—The previous experience of Mrs. Florence Brough Law, the new matron of Devon Hospital, was, we are informed, incorrectly described by the Secretary. She was not Lady Superintendent of the Kent Nursing Institution, Bromley, but Superintendent of the branch home of that institution.—A wreath was sent from the Trained Nurses' Institute at Weymouth to Windsor. It was composed of white flowers, mostly lilies of the valley, and the card, which was tied on with broad white ribbon, had the following inscription: "In ever-loving memory of our beloved Queen Victoria, from her sorrowing and loyal subjects the Superintendent and nurses of the Weymouth Trained Nurses' Institute."—The latest report of Sister Edith Ward from Pretoria is that she is "still seriously ill."—The nurses at the Eastbourne Homœopathic Convalescent Home are wearing on the left arm as mourning badges a piece of black corded ribbon, three inches wide, with a crown and "V.R." beneath, embroidered in gold silk.—We are asked to state that the floral harp which was sent by the matrons and nurses of Dublin at the funeral of Queen Victoria was quite distinct from the floral tribute sent by the matron and nurses of the Adelaide Hospital, Dublin.—Some more nurses left for South Africa on Monday last in the *Nubia*, including Miss S. Schliemann, who was trained at Ancoats Hospital, Manchester. She was subsequently charge nurse at the North London Hospital, Hampstead, sister in the children's ward at Ancoats, and, after doing private nursing for the Blackheath Institute, has, since last August, been at the Military Hospital, Devizes.—The proprietor and committee of the Nurses' Club, St. Andrew's House, Mortimer Street, W., have decided that matrons and nurses following their profession, and residing more than 50 miles out of London, will be eligible for membership at the reduced annual subscription of 5s. The entrance fee will be 21s., and no reduction of the 5s. is to be made in favour of members joining the club after January in any year.

### Lectures to Nurses on Medicine and Medical Nursing.

By NORMAN DALTON, M.D. London, F.R.C.P., Physician to King's College Hospital.

(*Continued from page* 236.)

### IV. FEVER.

### THE PULSE—RIGORS—THE COMPLICATIONS OF FEVERS.

THE "pulse" during the pyrexial state is more frequent than normal, and may be 80, 100, 120, or 140; or it may be, especially in children, too rapid to be counted. If the beats be very rapid, the artery usually feels small, because it is underfilled with blood. If the pulse be a "bounding" pulse, that is, if it be quick, full, and strong, the fever is called a "sthenic" fever; while if it be quick, but soft and easily compressed by the finger, the fever is called an "asthenic" fever. Most fevers, such as small-pox, scarlet fever, &c., are sthenic at first, except when they occur in old or weakly persons; but if they last any time, the pulse changes in character, and the type of the fever becomes asthenic. Typhoid fever and diphtheria, however, are often asthenic from the first. On the other hand, in acute pneumonia the fever is not only sthenic at first, but the pulse may remain full and strong up to a few hours before death. This is an important thing for a nurse to know, for she may be alone with her patient when the pulse changes from strong to weak, and can, at least, give the alarm.

It may be mentioned here that there are many other conditions besides fever in which the pulse becomes quick. The condition of the "skin" in fever is very important, quite apart from the fact that in the specific fevers, such as measles, &c., a rash is often present. As a rule the skin is hot and dry, but this is not invariably the case. For instance, in rheumatic fever the skin is bathed in a profuse, very acid perspiration, even when the temperature is 102° or higher. Again, in typhoid fever, in septic fevers, and in the fever of tubercular diseases the patient may often sweat, in some cases profusely, but in these diseases it will usually be found that the sweat breaks out when the temperature is remitting; that is, when a fall of two or three degrees is taking place; and that the sweating ceases when the temperature begins to rise again. Nurses should always note and report whether perspiration takes place or not. In some diseases, such as acute pneumonia, typhus, malaria, some cases of influenza, &c., the outbreak of sweating may be the earliest indication that the temperature is falling to normal, and that the patient will recover; and such perspiration should be encouraged by covering the patient with blankets. It must be confessed, however, that the occurrence of sweating is not such a constantly favourable sign as it used to be considered by old practitioners.

We may now consider the shivering fit which ushers in so many fevers. It is called a "rigor," and is probably caused by the first rush of the poisoned blood through the capillaries of the brain and spinal cord. It varies very much in degree. Sometimes, as in measles, mild cases of influenza, typhoid, &c., the patient only feels chilly, and complains of little cold thrills running down his back. In typhoid there is often a recurrence of these chilly feelings for two or three nights before other symptoms attract attention. In typhus, pneumonia, scarlet fever, &c., there is usually one very distinct attack of shivering, often accompanied by vomiting, and in smallpox and malarial fever the patient may shudder so strongly as to shake the bed, the teeth may chatter, and the skin become "goose-skin." In children the rigor may take the form of a convulsion. Nurses seldom see the rigor which begins a fever, because it is usually over before they are called in; but in lying-in cases a shivering attack may be the first sign of a fatal puerperal septicæmia. Rigors may occur during the course of a disease as well as at the beginning, but this is not common except in the case of pyæmia. In this disease the occurrence of a rigor usually means that a fresh part of the body, such as the liver, the lungs, &c., has become infected, and that the formation of an abscess in it has begun; and in erysipelas it usually means that a fresh piece of skin is attacked and will become inflamed. Rigors are not infrequent in that inflammation of the lining of the heart called septic endocarditis, which so often comes under the notice of the physician. If a rigor should occur in the course of typhoid fever, smallpox, or the other fevers it usually means that some complication such as pneumonia, peritonitis, &c., has set in. So a rigor is always a serious sign, and any complaint of chilliness on the part of the patient should immediately attract attention, and the temperature should be taken in the rectum. For, unless the patient shonld die in the rigor, which is extremely rare, the temperature nearly always rises a great deal after it, and the rise in the deep parts begins while the skin is still cold. During the rigor hot bottles should be put into the bed, the patient should be covered with blankets, and hot drinks with brandy should be given.

During the course of many febrile diseases the patient is very liable to be attacked by some new affection which is then called a "complication" of the original disease. There are some complications which are liable to occur in every fever which is at all severe. For instance, in any disease in which the temperature remains at 102° or 103° for a number of days, there is a great tendency for bronchitis or pneumonia to supervene and for the kidneys to be so affected that albumen appears in the urine. On the other hand, there are other complications which are so common in one particular disease and so uncommon in others that they may be considered to be specially connected with that disease. For instance, in rheumatic fever inflammation of the lining membrane of the heart is exceedingly likely to occur, and there is a special kind of inflammation of the kidneys which is associated with scarlet fever. Nurses should know the complications which are likely to occur in any disease which they may be nursing, and in the lectures on the specific fevers they will be mentioned. It is not always easy to draw a line between a symptom of a disease and a complication. For instance, diarrhœa is a symptom of typhoid fever, because it occurs in nearly all cases; but if the motions were as frequent as ten or twelve times a day, the diarrhœa may be considered to be a complication. Again, two diseases may exist in one patient at the same time without one being strictly speaking, a complication of the other; for instance, diphtheria and scarlet fever not unfrequently attack an individual simultaneously.

(*To be continued.*)

### Wants and Workers.

SISTER DOROTHY POTTERTON warmly thanks those kind friends who sent her THE HOSPITAL last week. One kind friend has promised it to her each week.

### To Nurses.

**WE invite contributions from any of our readers, and shall be glad to pay for "Notes on News from the Nursing World," or for articles describing nursing experiences, or dealing with any nursing question from an original point of view. The minimum payment for contributions is 5s., but we welcome interesting contributions of a column, or a page, in length. It may be added that notices of entertainments, presentations, and deaths are not paid for, but, of course, we are always glad to receive them. All rejected manuscripts are returned in due course, and all payments for manuscripts used are made as early as possible at the beginning of each quarter.**

### Nursing at the Workhouse Infirmaries.

### I.—ST. GEORGE'S UNION INFIRMARY, FULHAM ROAD.

By Our Commissioner.

The series of articles on nursing at the workhouse infirmaries, which it is intended to publish at intervals in The Hospital Nursing Mirror is appropriately opened with the account of an inspection of St. George's Union Infirmary, Fulham Road, for it was in respect to this infirmary that, more than a year ago, complaints were made in these columns on several matters of importance. Since then various improvements have been recorded, but, so far as nurses are concerned, the greatest of all is now in course of progress. I refer to the new Staff Home, which is to be opened in the spring, and which, on the occasion of my visit, the other day, was so far advanced that the matron, Miss Hampson, was able to show me over a number of the rooms, and to afford me an excellent idea of the accommodation provided.

### The New Home.

There are no less than 145 rooms in the new building, which runs along one side of the infirmary. Of these 131 are bedrooms. There are separate sitting-rooms for the assistant matron, the home sister, the head nurses, and the probationers; and in addition to a large messroom for the nurses, there is one of excellent size for the servants. Every nurse has a fireplace in her bedroom, and the rooms of the night nurses are on a floor shut off from the rest of the establishment. The number of bathrooms and lavatories is adequate, and there are three staircases and a lift. The corridors are heated by steam, the whole of the place is lighted by the electric light, and, as in the infirmary, there is telephonic communication. The new blocks include a laundry of three departments—one for the linen of the staff, another for the general linen of the patients, and a third for their foul linen; also an Isolation Building; Receiving rooms, Observation wards, and bathrooms for patients; a new mortuary; a new patients' clothing stores; a new dispensary; a new sewing store for the matron; a cook's larder; and large stores for the steward.

### The Mixed Wards.

As we passed into the wards, I noticed that the children are still distributed among the adult patients, and the matron informed me that there is no intention to alter the arrangement.

"The nurses," she said, "like it, and make pets of the children. Moreover, we do not think it wise to segregate children."

"What are the means of communication at night?" I asked.

"There are electric bells, and a telephone is now in the ward, so that the nurse can telephone the porter at any moment to send the night superintendent."

### The Question of Meals.

At present the nurses have a commodious temporary messroom, and, as we looked into it, flowers were being placed on the tables. The nurses themselves, by means of a weekly payment, keep the flowers going; and the housekeeper, who looks after the issuing of the stores, helps the assistant-matron and home sister to preside at the meals. "So that," as the matron remarked, "there is always some responsible person in attendance to see that the meals are properly served." As to the cuisine, the matron took me into the kitchen, and we briefly interviewed the cook in her very lofty and airy domains. With pardonable pride she showed me her well-stocked larder, and the preparations for ensuing meals indicated no lack of variety, and no stint in quantity, with fruit as a feature. From the kitchen we went into the room where the home sister was holding her class for junior probationers, and then, returning to the matron's office, Miss Hampson expressed her readiness to answer any questions as to the training.

### The Staff.

"What is the full strength of your nursing staff?" I inquired.

"There are 14 sisters, 42 staff probationers, and 35 junior probationers, 91 in all. As doubtless you know, St. George's Infirmary was the second in London to start a training school. The period of training is for three years, and candidates are on trial for two months. At the expiration of that time the medical superintendent and matron have to report separately whether the candidate is a fit and proper person for training."

"If accepted what form does she have to sign?"

"As follows: 'Having after a trial of two months been accepted upon the conditions specified in the regulations for probationers, I hereby agree to remain in the service of the guardians of St. George's Infirmary for the full period of three years' training.' At the end of the period, if she pass the examination satisfactorily, she gets her certificate, which is signed by the Chairman of the Board, the medical examiner, the medical superintendent, the medical officer, and the matron. If after twelve months she breaks her engagement, she may receive a certificate of character only, signed by the medical superintendent, the chairman of the visiting committee, the clerk to the guardians, and the matron."

### Uniformity of Work.

"Would you like to say anything about the duties?"

"Uniformity of excellence of work is aimed at. When a candidate commences her probationership she is handed a list of the duties of the nurses which has been approved by the visiting committee but further than this I have the daily routine prepared for the sisters and ward nurses. Every probationer who is in charge of a ward, and is in her second or third year, is known as a staff probationer. Each sister is presented with a report sheet for every probationer under her, and she fills it in and returns it when the probationer is taken away. By this method I know exactly what each nurse has been doing."

The matron courteously offered me the daily routine to which she referred. The directions given are of an exhaustive and carefully thought-out character, and the list of "weekly duties to be arranged for such days as each sister finds most convenient," as well as the "notes" must be distinctly helpful.

### No Overworked Nurses Now.

It will be remembered that complaints were made of the insufficient staff, resulting in overwork, and in answer to a question the matron said—

"This was remedied last June, when a night superintendent, six head nurses, and ten probationers were added to the staff. A previous addition of ten probationers was made in November, 1897."

"What is the number of beds?"

"Seven hundred and seventy-six; there is, therefore, with ninety-one nurses, about one to eight patients. This is a medium average—about the same as at St. Marylebone."

"You had only one night nurse to two wards?"

"Now we have two—one for each ward; in fact this is essential, each ward being on a single floor. There are three extra night nurses on duty to take nights off, and for special cases."

Off Duty and Recreation.

"How many nights off do the night nurses get each month?"

"Two. When the additions were made to the staff in June, I was able to draw up fresh time-tables for both night and day nurses. The staff is now quite adequate, and all the nurses get a very good time off."

"May I ask what you call a good time?"

"Every day nurse has one whole day off duty each month, as well as 10½ hours each week away from wards during duty hours, exclusive of time for meals, for rest, study and recreation. This is exclusive of Sunday leave. The night nurses get about the same. As to holidays, the head nurses have three weeks, and the others, unless they have been here three years, a fortnight."

"You mentioned recreation. What provision is made in that direction?"

"There is a concrete quadrangle for the use of the nurses, who can either cycle, skate, or play tennis. The present amusement is skating. They can obtain so much exercise in the quadrangle that they really do not need to go out if they prefer to stay in when they are off duty."

The Class Instruction.

"What is the nature of the teaching imparted by the home sister whose class I saw just now?"

"The home sister deals with practical work. She takes the junior probationers over the three doctors' lectures, and instructs them in such things as bed-making, bandaging, the use of the thermometer, medicine measuring, poultice making, fomentations, and the preparation of special foods. She attends the doctors' lectures with them, and a very special feature of the training is that they write all the lectures and she corrects the books."

"She must have a large number to correct?"

"At least 200 a week. But we make a most thorough business of the class instruction. The medical superintendent and two assistant doctors lecture on anatomy, physiology and nursing, and attendance is required at the classes through the year, except in the holiday season, from June to September, when we always take the junior probationers over the ground-work."

Conduct Counts.

"Is the[illegible] an outside [illegible]ation?"

"Yes, at the end of the third year; and the matron's report for conduct and work counts at that examination. You may have a clever probationer who can pass the written examination well, but who is not by any means a skilful or trustworthy nurse."

"I should like to say," continued the matron, "that we exercise great care in the selection of candidates, and endeavour to get those who seem best fitted, by education and physique, for our training."

"Both the home sister and the senior night superintendent have been here for twenty years."

"And yourself?"

"I was appointed in May 1897. I was trained at St. Thomas's Hospital, and was afterwards sister at St. Marylebone. In August, 1894, I went to Cork Street Fever Hospital, Dublin, as assistant matron, and in April 1895 I became lady superintendent. There I remained until I came here."

A Universal Curriculum Wanted.

"Now, with all your experience, I should like to know your views on the general question of the administration of workhouse infirmaries,"

"We want a Central Board of Management, who would endeavour to obtain uniformity. I think we ought to aim at a universal curriculum, with a syllabus of instruction, so that one medical superintendent should not be teaching one thing while a second is teaching another. If they all taught the same thing, we should have a Board of Medical Examiners, and then every nurse from every infirmary would stand the same chance of promotion. With a universal curriculum and expert inspectors—this is a point of great importance—nurses would get uniform rates of remuneration for similar services, which, in existing circumstances, is impossible,"

"In conclusion, I should like to point out that it seems to me most desirable that guardians should engage a medical superintendent and a matron of another infirmary training school to conduct their own examination of candidates, the doctor taking anatomy and physiology, and the matron the practical nursing. The practice of engaging a hospital physician who has not been engaged in teaching nursing has been, in my experience, attended with disadvantages."

And then, armed with much literature, I came away from St. George's Infirmary, satisfied that the matron has a high sense of her responsibilities, and is sincerely anxious to promote the interests of the nursing staff.

### How they Received the Sad News at Vancouver.

By SISTER FRANCES.

January 22nd, 1901.

We have just received the cablegram giving the sad news of the death of our beloved Queen, and how far away we feel! Yet the message was received 15 minutes after its dispatch from London. Our rector, Mr. Fiennes-Clinton, at once tolled the church bell, so that the residents should know, for we had been watching and hoping, and the telephone had been going night and day. There are many English people here, and we all cling so to each other. On Sunday, the 20th, after evensong in St. James's Church, we had a short special service, ending with "God save the Queen;" the large congregation joining in could not help realising that it might be the last time, and so it proved. On the evening of the 22nd, the rector announced in the evening papers that there would be a special service at 8 p.m., and at that hour our church was filled—all classes and from all parishes. We had the service for the dead and it seemed a comfort to be able to meet together and do something. Mr. Fiennes-Clinton must have felt the strain very much, for, apart from his own feelings, so many came—all with some badge of mourning on—to talk and ask what they could do, how they could help. We, the nurses and myself, draped our choir stalls. The altar had a black frontal with a super-frontal of white lace, only used on special occasions. I cabled my respectful condolences to Osborne and in a few short hours received the following reply:—

*Osborne*, 23*rd*.

To Sister Frances, St. Luke's Home, Vancouver.

"Princess of Wales' heartfelt thanks for kind sympathy."

How I prize and treasure the gracious kindness of her who in the midst of her own grief could think of one so humble! It is expected that the "Sons of England" will assemble before the church service on February 2nd in the Victoria Jubilee Ward where the picture of our beloved Queen hangs, which she graciously sent for the ward and which is the only photograph in the West. Our mayor has ordered everyone to go into deep mourning for one month. All our public buildings are draped and all gaieties are stopped. The first places to close when the news came were the saloons, followed quickly by the theatres. I think those who may have friends out here will like to know that we were not one whit less loyal because we have made our home in a far-off Western city.

### Canon Scott-Holland on Nursing.

By Our Own Commissioners.

This year, the annual meeting of the District Nursing Association for Hammersmith and Fulham was held in the Lyric Theatre, Hammersmith, by kind permission of the manager, who was himself present, and who gave a donation to the Nursing Fund. Mr. W. J. Bull, M.P., was in the chair, and he was supported by the Rev. Canon Scott-Holland, Mrs. Scharlieb, M.D., Mrs. McCallum, the Mayor of Hammersmith, the Revs. E. G. Wood, Joseph Dixon, and Michael Adler, Mr. J. M. Levy, Mr. G. Coffin (representing the Amalgamated Society of Railway Servants, which is a yearly subscriber to the Nursing Fund), and Doctors Wells and Gunton Alderton.

The eleventh annual report showed that the total number of cases nursed during 1900 was 1,106, 20,509 visits were paid, 3,064 minor dressings, &c., were done in the Board Schools. Cases had been sent in by medical men and hospitals, 515; by clergy and district visitors, 226; by the Charity Organisation Society, 9; by the Board Schools, 65; and by patients' friends, 218. Subscriptions, donations, patients' small payments and probationers' fees, amounted to £673 13s. 5d.

### Apostles of Health.

Mrs. Scharlieb, in moving the adoption of the report and balance-sheet, alluded to the interest always taken by Her late Majesty Queen Victoria in district nurses, and recalled her gracious invitation of the Queen's Nurses to Windsor Castle. The work of a district nurse, Mrs. Scharlieb said, was of a very wide-reaching character; it was much to attend to the bodily needs of their patients, but they were, besides, missionaries and apostles of health. They were able to impart most valuable information where it was most needed. The essentials of health were fresh air, pure water, and good sanitation.

After some further speeches,

Canon Scott-Holland addressed the meeting.

### Nurses and the Housing Question.

"In the first place," he said, "nurses can impart some knowledge of sanitation, in the face of the awful ignorance which reigns amongst the poor. There is a firm belief that anything in a bottle is medicine, and that all medicines may be taken indiscriminately, then and there. That is a belief I share entirely myself. (Laughter.) I should like to be able to speak with all my heart as to the joy of nursing poor people in their own homes. No one can believe more fully than I do in home life; and I can picture a poor man being nursed in his humble home. There is a certain irony in the matter. I read only the other day of a man suffering from the most horrible complaint, gastric ulcers; there were ten other people living, eating and sleeping in this 'home.' That is the sort of home in which we expect all the moral virtues to grow up! At the beginning of last century there were 900,000 people in London; at the end of it, there is a population as large as that living and sleeping under the soul-destroying conditions of a one-roomed tenement. One room in which at the very least two or three people live, sleep, work, wash, cook, do everything, are sick, and die! When we speak of nursing the poor in their own homes, let us think what we are doing. There are 26,000 people living six in one-room tenements. And this is not only in the East End or in the centre of London; it is here in Fulham that you have your inspector reporting that eight or ten people are living in one room; in one case four adults and six children in a single room, 10 feet by 10 feet, 800 cubic feet, 'sufficiently large for two people. That is where home sick-nursing has to be carried on. Homes? Just think what sickness means in such homes! Remember what we find when we are sick. Instead of a wretched little cabin with screaming children and all the filth and noise and hustle, we have quiet and sympathy and everyone ready to wait on us. When the Socialists came to St. Paul's Cathedral and the preacher said, 'we are all equal in our sickness,' a voice came from beneath the dome, 'We are not; you go to the South of France.' I know of no time when the division between the rich and poor is so clearly marked." Canon Scott-Holland illustrated this distinction by a story from Tolstoi. A carriage is conveying to her home a countess who is dying. She is taken into the midst of the greatest comfort and quiet, to be tended by skilful hands. The same groom and stablemen who have travelled with her go back to the stables, where an old man lies dying in a back room amid squalor and misery and noise. These are the words he hears from those round him as they discuss what each will have of his poor possessions: "Old man, what are ye so long dying for?"

### Bridging the Gulf.

"But," said the Canon, "we can do just a little to relieve this difference. We can reach out kindly hands and tenderness to those who suffer; we can bridge across the gulf a little way and bring some touch of home into the worst hovels in London by intelligent and skilful kindness. That is why we ask you to put your very best effort into a work of this kind. Surely, there is something to be learnt from the way in which our great Queen came forward in the matter, feeling, in her sympathetic heart, that however wide the gulf might be, something could be done through the tenderness of nurses. And she did that something. It is a little thing which can be actually done whilst we are still talking about the great social evils that surround us—something to be done with a clear conscience, something absolutely good. Our Lord, though His heart was set on the work before Him, still found time, by the way, to do the one kind thing which was most evidently to be done. It is always, as it were, the one thing that reaches Christ. All perplexities are left behind. Is it their own sin, their own fault? It does not matter. They are there and they are sick, and I can help and cure them. There are no more questions to be asked. That is enough. With full power of heart and brain we are to do just the thing that lies to our hand. That is what we want you to do, to look over the great piteous woes of life, and to say, "Here is a thing I can do; here is a thing which day by day is a witness that, in spite of all the evil in the world, God is here, The poor, in the only homes they know, shall at least be sure that there are patient hearts and skilful hands ready to preach to them the great truth which is the only truth in life, the truth that all this world seems to obscure, that "Love is life, and life is Love."

### Minor Appointments.

Lewisham Union Infirmary.—Miss Ellen Lanoa McAvoy has been appointed ward sister. She was trained at St. Bartholomew's Hospital.

Portsmouth Workhouse Infirmary, Milton.—Miss Hannah Hollands has been appointed charge nurse. She was trained at Fulham Infirmary.

Middlesbrough Union Infirmary.—Miss Annie Elizabeth Cawood has been appointed charge nurse. She was trained for one year at the Fever Hospital, Blackpool, and for three years at the Union Infirmary, Middlesbrough.

Bridgwater Infirmary.—Miss Mary T. Webster has been appointed sister. She was trained at North Devon Infirmary, Barnstaple, and has since been assistant and district nurse at Chorley Cottage Hospital, sister of female wards at Macclesfield Infirmary, and sister of male wards at North Devon Infirmary.

Swansea Hospital.—Miss E. G. Holmes and Miss L. M. Rovedino have been appointed sisters. Miss Holmes was trained at the Queen's Hospital, Birmingham, and has since been sister at the Ear and Throat Hospital, Birmingham. Miss Rovedino was trained at the Hospital for Sick Children, Great Ormond Street, and the London Hospital. She has since been staff at the London Hospital.

### Echoes from the Outside World.

### AN OPEN LETTER TO A HOSPITAL NURSE.

NURSES across the seas will rejoice to learn that the rumour which pessimists set afloat directly upon the death of Queen Victoria, that the Royal visit to the Antipodes would necessarily be abandoned, is without foundation. The late Queen wished the visit to take place, and the King is glad that it should be so, but the Colonial Secretary adds just a warning note that "His Majesty is confident that, in making arrangements for the reception of the Duke and Duchess of York, the people will not fail to recognise the sad circumstances under which the visit takes place," consequently many festivities will be curtailed or abandoned; but the fact remains that the Heir Apparent and his wife will leave England in March, and return home by way of Canada. It is a matter of congratulation as far as Australasian trade is concerned that all the light fabrics which were long ago ordered from England will not be wasted, for the period of general mourning will be almost over by the time the Royal couple reach the Antipodes, and colours will be again permissible. Perhaps it is in order to keep as much as possible to the original scheme that the King's son will be styled Duke of Cornwall and York during his absence, the title of Prince of Wales only being bestowed upon him on his return, for which at present, of course, no date is given. But one thing is certain. Those people who were rash enough to say that the King's coronation would take place as early as July are utterly wrong, for it would be an impossibility without the Heir Apparent. Even his little son, who so pathetically represented his father at his great grandmother's funeral, when his father was absent from illness, now happily over, would not suffice upon such a grand occasion, although he will probably be there in the more humble rôle of the third generation.

LAST week I wrote about the funeral of a great Queen, who passed away from us after a long and noble career, well stricken in years. This week my pen runs naturally to the marriage of a young Queen who, though she has grown up amongst her people, has only been upon the throne a couple of years, and therefore is almost at the commencement of her Royal career. As I read of the preparations which were being made in Holland for the joyous occasion of Queen Wilhelmina's wedding, I could not help feeling full of sympathy for her that all the European world, who at any other time would have been deeply interested in her doings, should have been so wrapped in gloom that there was little heart left for rejoicing, nor even leisure to think of wedding presents. As yet I have seen no mention of England's offering, and am the more anxious to hear of it because I feel so sure that had our own dear Queen lived the message which she would have sent with her gift to the young bride would have been so kindly and so encouraging. The commencement of their lives, too, is so similar. The Kaiser, I see, presented a magnificent Dresden service, and Mr. Kruger—a thimble from the Paris Exhibition. The Dutch Colony in London have decidedly chosen of the best the land could produce, a Royal Worcester dinner service, of which the dishes were hand-painted by Sir Laurence Alma-Tadema. The City of Amsterdam gave their Sovereign a golden coach, surmounted by a splendid scroll emblazoned with the Royal Arms.

As to the Queen herself, all one hears of her makes one realise how exceedingly winsome she must be. Even the stories told of her childish escapades make her the more lovable. The one occasion when she tried to overawe her mother by describing herself as the "Queen of Holland" and failed to gain an entrance to the apartment till she changed her demeanour and admitted "I am your little daughter Wilhelmina, and I wish to speak to my mamma," or the other when her English governess offended her and found, in consequence, that in the next map of Europe which her pupil penned England had been entirely left out. The most pathetic incident is that related of the Queen and her dolls. Once when she told her troubles to one of them she became irritated at the smiling and unsympathetic face it continued to display, and exclaimed angrily, "If you are so horrid, I'll make you a Queen and then you'll have no little boys and girls to play with." Her education was most carefully superintended, and though she is still so girlish and natural, she is quite alarmingly clever. She speaks French, English and German perfectly, has a knowledge of Russian, and has even acquired some smattering of the Malay tongues spoken in her East Indian possessions. Her course of studies has included physics, chemistry, political economy, jurisprudence, navigation, and military tactics, and she is a splendid horsewoman. Her mother has always been most particular that her body should be developed as well as her mind, and the physical exercises which were part of her daily routine have done much to make her the strong healthy girl she is. As to the bridegroom, he is—or was—a German, consequently the Dutch people were disposed not to like him; but he is their "Willemyntje's" choice, therefore they are anxious to learn and look upon him favourably. He stands three inches higher than the Queen, though she is fairly tall, and it is reported that on his wedding day he looked very pale and very youthful, notwithstanding his four years' seniority. His position is rather a trying one, and he will need some tact to steer clear of difficulties. In civil life he is the husband, and the wife has promised and is bound to obey him; in State life it is she who is the head and he is to obey her. But love, which is said to make the world go round, will probably help also to keep the little bit of it known as the Netherlands in comfortable rotation.

THE marriage service was simple in the extreme. Two verses from the Book of Ruth as an anthem, a sermon, a hymn which both the Queens sang, the bridegroom holding the paper for his bride, two short questions to the contracting parties, with replies, an exchanging of rings, a benediction, and the knot had been tied. There were no bridesmaids, and no one gave the bride away. Her dress was magnificent, silk and silver, pearls and diamonds, all seemed worked into a harmonious whole, and the severe simplicity of the way it was made, the bodice being low and sleeveless, showed to perfection the roundness of the girlish figure. The Queen carried a bouquet of orchids and orange blossom, sprays of the latter flower being interwoven in the diamond tiara on her head. By a strange chance Dr. Leyds would have sat next the English Minister had not the Portuguese representative been between. The people assembled in great crowds to wish their Queen happiness, and the streets looked quite festive with fir trees transplanted from the woods of Scheveningen, hung with orange and white ribbons, and united to each other by garlands of green. Everyone wore a knot or flower of orange colour out of compliment to the reigning house.

### Everybody's Opinion.

SO-CALLED TRAINED NURSES.

"M. M. M.," Liverpool, writes: The case I gave in my letter of January 26th is far from being an isolated case. I know of far worse cases, and am sorry to say it is only one of many that has taken place through the same institution, of which I am quite prepared to give the name.

INFECTIOUS DISEASES.

"The Superintendent" of the London Association of Nurses, 123 New Bond Street, writes: Our notice has been drawn to an enquiry in your columns asking for information as to a home where children of the upper classes can be received and cared for when suffering from infectious illness. For many years we have had such a home in connection with our Association, and we have found it meet a very real need on the part of the public.

NURSES AND VISITORS.

"Indignant" writes: It was with great disgust, yet also with a slight feeling of amusement, that I read in "The Hospital" Nursing Mirror of February 2nd, an account of a meeting held in Eaton Place, anent a scheme for visiting the sick in our hospitals and infirmaries. Most decidedly the majority of ladies and gentlemen who spoke showed an utter ignorance of the wards and nursing in our institutions. I, as a nurse in one of the largest and best workhouse infirmaries, was justly indignant with the person who spoke of us as "the commonest description of pauper." It is true that in some of the smallest workhouse infirmaries the inmates help the nurses; but in no case are the nurses themselves paupers. One speaker gives it as her opinion that visitors should go in their best clothes. I think they would soon give up that idea were they to hear the remarks passed on them by the patients as soon as their backs are turned. It is hardly the thing to parade one's wealth before poor sick people who have not a penny in the world to call their own. It only makes them more bitter against the fate which has given them so little whilst others have so much. May the would-be visitors give up their scheme, and parade their best clothes and their latest head-gear before the eyes of their own personal friends. Nurses have more serious matters with which to occupy themselves than "the latest thing in hats."

TRAINED NURSES FOR CRIPPLED CHILDREN.

"E. B." writes: I was much interested in reading Mrs. Humphry Ward's address to nurses in the Mirror of January 26th, and fully appreciate her kind thought for the little crippled children, for they of all others need the care of a trained nurse who knows too well that if such cases are not properly handled much needless pain is caused. The work itself would be interesting to one who was fond of children, and would, I am sure, be willingly undertaken by the nurses, provided, of course, a fair remuneration were offered for such services. "Nurses over forty years of age," as Mrs. Ward says, "have gained much valuable experience, and are often glad to give up ordinary nursing"; but it would hardly be to their advantage to settle down on £50 per year for about 50 hours per week—the anxiety and difficulty of perpetually cutting down expenses in order to make ends meet would be too trying, and the nurse's spirits would soon flag. Little crippled children recognise too often their sad affliction of not being like others, and they should have as much brightness round them as possible, to make up for their sad lot, and the nurse could hardly be expected to be bright and cheerful under such straitened circumstances. In engaging a nurse it seems to me that the remuneration from her point of view is not duly considered, the great idea usually being to obtain her services as cheaply as possible. Now in London a professional nurse cannot provide herself with board and lodging, &c., laundry and uniform, for less than 30s. per week; then in addition to this she needs a holiday now and again, which, as we all know, costs something; and every sensible, thoughtful woman wishes to save a little for old age. It really is sad to think that in rich, powerful England a woman's work should be so cheap. Would it not be well for those in authority to sometimes consider those memorable words of Cato, the Roman Censor, "It is better that the many should have silver in their pockets than that the few should have gold"?

SISTERS AND PROBATIONERS.

"M. W. W." writes: I, like "An Old Hand," read Miss Twining's letter with great interest, and would like to give my own case as an example. I entered a large London hospital as probationer and unfortunately was put under a sister devoid of all feeling towards new probationers. For three months my life was a burden, and this sister's verdict of me was that I was mistaking my vocation and would never make a nurse; so after three months of utter misery matron asked me to leave. After a few months' rest I entered the profession again as probationer, with very happy results, and now hold a very good post as district nurse. Those under whom I have worked speak most favourably of me. Therefore I think it advisable to let a probationer work under more than one sister before giving up all hope of her ability.

### Appointments.

Chelsea Infirmary.—Miss Eleanor C. Barton has been appointed matron. She was trained at St. Bartholomew's Hospital and the Royal Hants County Hospital. For more than three years she held the post of superintendent of the Nurses' Home at Chelsea Infirmary, and for the last nine months she has been matron of the Convalescent Home for Children connected with that institution.

Bloomfield Retreat, Dublin.—Miss Margaret Haughton has been appointed lady superintendent. She was trained at the City of Dublin Hospital, and has since been matron of Barrington's Hospital, Limerick.

Shotts Fever Hospital, Lanarkshire.—Miss Iza Bell has been appointed matron. She was trained at the City of Glasgow Fever Hospital, Belvidere, and also at the Royal Infirmary, Glasgow, in both of which institutions she was in charge of wards.

Atcham Workhouse Infirmary, Shrewsbury.—Miss Alice Edgcumbe Chevallier has been appointed superintendent nurse. She was trained at Crumpsall Infirmary, Manchester, where she was also ward sister. She has since been sister at Prescot Union Infirmary, and sister at Selly Oak Infirmary.

Horsham Workhouse Infirmary.—Miss Rose Andrews has been appointed superintendent nurse. She was trained at Greenwich Union Infirmary, and has since been assistant nurse at Mile End Union Infirmary, ward nurse at St. Saviour's Union Infirmary, and staff nurse at Greenwich Union Infirmary.

Hitchin Workhouse Infirmary.—Miss Grace Mary Lloyd has been appointed superintendent nurse. She was trained at Sunderland Workhouse Infirmary, and has since been nurse at the South-Eastern Fever Hospital, New Cross, charge nurse at Lambeth Infirmary, and head nurse at the Lewes Union Infirmary.

Infectious Diseases Hospital, Wallsend.—Miss Emily Morgan has been appointed nurse matron. She was trained at the Royal Infirmary, Newcastle-on-Tyne, and has since been nurse at the Thomas Knight Memorial Hospital, Blyth, and Eston Sanatorium, near Middlesbrough. She holds the Glasgow certificate for midwifery and monthly nursing.

Tunbridge Wells Eye, Throat, and Ear Hospital.—Miss Jane Ord has been appointed matron. She was trained at Guy's Hospital, and holds the silver medal for five years' service. Subsequently, she was attached to the Nurses' Corporation and the private nursing staff of Guy's Hospital, and was sister of the women's wards at the Tunbridge Wells General Hospital and sister at the Tunbridge Wells Eye, Throat, and Ear Hospital.

### The Great Northern Central Hospital.

INTERVIEW WITH THE MATRON.

A PARAGRAPH having appeared in one of the daily papers headed "Typhoid Outbreak! Cases in a London Hospital kept in Check," our representative called on the matron of the Great Northern Central, the hospital in question, to enquire into the correctness of the statements.

"It is quite true," said the matron, "that there is typhoid in the hospital, and that we have closed two of the wards. But as we should have done this in about a fortnight's time in any case for spring cleaning, the matter is less serious than would appear. We should in any case have had some extra painting done this year, and we intended taking the opportunity of emptying these wards while our staff was short."

"Was it short on account of typhoid?" asked our representative.

"No. Three of the nurses had mumps, and were consequently off duty. To avoid the disadvantages of getting in strangers as substitutes, we decided to have the necessary cleaning done during their absence from the wards. Then a case of typhoid appeared, and it was still more necessary to avoid having fresh nurses in, who might be more likely to take infection. We thought at first that it was a case of scarlet fever, and the nurse was sent to the Fever Hospital. However, it turned out to be typhoid, but of a very mild form."

"And then it spread?"

"Three other nurses took it, and two maids, one of whom is giving us some cause for anxiety. But the rest are all going on very satisfactorily, and the form is happily a mild one."

"It has not spread farther among the staff?"

"No; the rest of the staff are very well, and we quite hope that in a few weeks everything will be right again. It is quite possible that while we are short-handed we may turn out two more wards for painting; if we do so it will not mean that we are closing the wards on account of infection. It is rather tiresome when a simple action of this kind is misunderstood by the public."

"It will only mean that it is easier to get on with the cleaning while some of the staff are away?"

"Exactly."

"The nurses and servants are being nursed in the hospital?"

"Oh yes; we have every convenience for typhoid: it is an illness we constantly have in the wards, so it is nothing out of the common for us. The only trying thing is that it is among the staff. They will be sent away when they are convalescent."

"And now is anything known as to the cause of the illness among the nurses?"

"No; its origin has not yet been traced. There has been a thorough inspection of the drainage system, which seems to be perfect. We came into this building in 1888, so it is comparatively new: I think the first patients were received on April 13th of that year. The hospital was opened by the Prince and Princess of Wales."

"Of course precautions are being taken to prevent the spread of the illness?"

"All the water and milk used in the hospital are being boiled."

"And visitors are being excluded?"

"They are. But this again looks worse than it really is, for of course no one expects typhoid to be taken by a casual visitor going into the ward where there are cases. I think perhaps we were just a little frightened at first, and took all the measures we could possibly think of to prevent its going any further. However, I hope soon we shall be quite free, and the staff at work as usual."

### Death in our Ranks.

WE learn, with regret, of the death, at the early age of 24, of Miss Janet Hooper, a nurse at Mill Lane Hospital, Liscard, Cheshire. Miss Hooper had been suffering from typhoid, but was convalescent, and died quite suddenly after complaining of feeling faint. She was very popular in the hospital, and her funeral was attended by her fellow nurses and a number of the medical staff.

GREAT sadness was caused at the General Hospital, Birmingham, by the death early on the morning of Sunday, February 3rd, of Nurse Margaret Rebecca O'Neill, after a short illness from enteric fever. Miss O'Neill, who was born in Belfast on February 4th, 1874, was trained at the Lewisham Infirmary for three years, and was afterwards nurse at Walsall District Hospital for a short time. On November 19th, 1900, she joined the staff of the Birmingham General Hospital, where she made many friends by her brightness and cheerfulness. On Tuesday morning a short service was held in the hospital chapel by Rev. J. H. B. Masterman in the presence of Mrs. and Mr. John O'Neill (mother and brother), Sir John Holder (Chairman of House Committee), Mr. John Phillips (Chairman of Nursing Committee), Dr. T. S. Wilson (hon. physician), the House Governor, seven residents, 49 members of nursing staff, including the matron, assistant-matron, and night superintendent, 22 wardmaids, and servants, Dr. Sedgwick, one of the house physicians, presiding at the organ. At the close of the service Mrs. and Mr. John O'Neill, the matron, night superintendent, four nurses, and the House Governor followed the coffin to the Warstone Lane Cemetery, Birmingham, where the committal prayers were offered by Rev. J. H. B. Masterman. A large cross of white flowers and violets from the matron and nursing staff, and wreaths from the Chairman of the Nursing Committee, Dr. Wilson, the resident medical and surgical staff and the House Governor, were placed on and around the coffin in addition to those contributed by the sorrowing relatives.

### Presentations.

NEWBURY DISTRICT.—Miss Freeman, district nurse for St. John's and Greenham, has been presented with a bicycle, a purse, and a book with the list of subscribers and an address signed by the inhabitants gratefully recognising her services. The idea of making this present originated amongst those who had most benefited by Miss Freeman's kind ministrations, and the response was so hearty that it resulted in the sum of £34 10s. being contributed by over 700 subscribers. The presentation is the more noteworthy as Miss Freeman is not leaving the parish.

PARISH HOSPITAL, PAISLEY.—Miss Halliday, who has been appointed superintendent of nurses at the Plymouth Union Infirmary, has been presented by the Parish Council of Paisley with a gold bracelet and sleeve-links in recognition of her services during a period of nearly eleven years. Miss Halliday was chosen to organise the new Parish Hospital in Craw Road and to superintend the first staff of trained nurses, and her services in the capacity of lady superintendent were warmly acknowledged by the deputation from the Parish Council who made the presentation.

BLACKBURN DISTRICT NURSING ASSOCIATION.—At the monthly meeting of the Blackburn District Nursing Association last Thursday, February 8th, Miss F. E. Nash was presented with a very pleasing recognition of the regard which the committee entertain for her. She is leaving her work in the town to be married, and the committee took the opportunity of giving her a wedding present, consisting of a case of fish knives and forks with carvers and a silver serviette ring. Nurse Nash has been a district nurse in Blackburn for two years, and during that time has won general esteem by her unfailing kindness to her patients, and by the conscientious manner in which she has performed her duties. As a token of the goodwill she had earned among her fellow nurses, they presented her with a silver-mounted bread trencher with knife.

### For Reading to the Sick.

"OUR FATHER,
WHICH ART IN HEAVEN."

FATHER of all, to Thee
With loving hearts we pray,
Through Him, in mercy given,
The Life, the Truth, the Way;
From Heaven, Thy Throne, in mercy shed
Thy blessings on each bended head.

Father of all, to Thee
Our contrite hearts we raise,
Unstrung by sin and pain,
Long voiceless in Thy praise;
Breathe Thou the silent chords along,
Until they tremble into song.

Father of all, to Thee
We breathe unutter'd fears,
Deep-hidden in our souls,
That have no voice but tears;
Take Thou our hand, and through the wild
Lead gently on each trustful child.

Father of all, may we
In praise our tongues employ,
When gladness fills the soul
With deep and hallow'd joy;
In storm and calm give us to see
The path of peace which leads to Thee.

*J. Julian, D.D.*

Oh to learn more truly to believe in the Fatherhood of God! God, my Father, not when I am good only, but when I am bad and reckless and wayward; God, my Father, in sickness when I am tossing on a bed of pain; God, my Father, when I am shunned, distrusted, suspected, hated; God, my Father, when all the hopes and promises and plans of my heart are crushed, shattered, shrivelled before my eyes.—*T. B. Dover.*

God the Father compassionates our infirmities as a Creator, who, while He made man in His own Image, formed him at the same time of the dust of the ground. God the Son compassionates our infirmities, as One who has taken our nature upon Him, and shared our circumstances; while God the Holy Spirit brings down the Divine sympathy of the Father and the Son to the succour of the individual Christian, and makes it a reality in our experience by the internal assistance which He gives us in our prayers.—*Goulburn.*

Let us take care of our conduct, and God will take care of our feelings, and make them fervent or dry as to Him shall seem expedient. In devotional exercises, what we have to look to is our intention. If we intend to do right, God knows the heart, and we have an Advocate with the Father for our ignorances and negligences.—*Dean Hook.*

O Lord, I know not what I should ask of Thee. Thou only knowest what are my wants, and Thou lovest me better than I can love myself. O Lord, give to me, Thy child, what is necessary, whatsoever it may be. I dare not ask either crosses or consolations: all that I shall do is to present myself to Thee. Behold my wants according to Thy mercy. . . . I adore all Thy purposes without knowing them. I entirely abandon myself to Thee. I have no more any desire but to accomplish Thy will. Lord, teach me to pray, and pray Thou Thyself in me.—*Fénelon.*

### Notes and Queries.

**The Editor is always willing to answer in this column, without any fee, all reasonable questions, as soon as possible.**
**But the following rules must be carefully observed :—**
**1. Every communication must be accompanied by the name and address of the writer.**
**2. The question must always bear upon nursing, directly or indirectly.**
**If an answer is required by letter a fee of half-a-crown must be enclosed with the note containing the inquiry.**

*Kingston Union Infirmary.*

(219) 1. Kingston Union Infirmary not being a recognised training school for nurses, what is the position of probationers already in residence ? 2. Will their certificates date from their arrival on the premises, or from the date when a resident medical officer shall be elected by the Board, and shall come into residence ? 3. If the latter, are not the services of these probationers being obtained contrary to the regulations of the Local Government Board ? 4. Under these circumstances, are they bound by their agreement to remain ? *A. E. F.*

The information you desire can only be obtained from the Local Government Board, Whitehall, S.W. The Board is the final arbiter as to what it will accept as a proper "training," and each case has to be determined on its merits. The question raised by A. E. F. is one of very great importance.

*Barbados.*

(220) Seeing that a head nurse has not been secured for the General Hospital, Barbados, I should be deeply obliged if you would tell me where to apply, what the climate is like, and what the difficulties are at the aforesaid hospital ?—*L. O. H.*

Apply to the secretary of the hospital. The climate is like that of other colonies in the West Indies. There is no winter. Some difficulties have been mentioned, but we cannot deal with them here.

*Massage.*

(221) Can you kindly inform me where I could have lessons in massage at Glasgow ?—*Bessie.*

The Secretary of the Society of Trained Masseuses, 12 Buckingham Street, Strand, W.C., would recommend you a reliable teacher in Glasgow.

*Age Limit.*

(222) Would you tell me if, at the age of 37, I am too old to be trained either in general or maternity nursing ?—*L. I.*

You are not too old to qualify as a maternity nurse, 40 years of age being the limit at most of the lying-in hospitals. But a general training after 35 can only be obtained as a paying probationer. Terms, at the Royal Free and the London, £13 13s. a quarter.

*Training.*

(223) Would you kindly inform me where I can get two years' training ? I have a certificate for one year, but find it of little use.—*Nurse Marie.*

Many of the special hospitals give a two-year course; for instance, the Poplar Hospital for Accidents, or the Cancer Hospital, Brompton. The Matron of the Hospital for Diseases of the Chest, Victoria Park, E., makes special arrangements with regard to this kind of certificate. Before definitely accepting any appointment write to the Local Government Board, and ask if your training, when complete, will qualify you for the post of superintendent nurse. The address is Whitehall, S.W.

*Sufficient Qualification.*

(224) Is a four months' training in maternity nursing and the possession of the L.O.S. certificate sufficient qualification to enable me to join a nurses' co-operation ?—*Energy.*

No. Not as a nurse. Most associations require a three years' certificate in general nursing as well as the L.O.S.

*Mental Nurse.*

(225) Are there any asylums in London or the provinces where a mental nurse over 40, healthy, active, with good personal character and some experience, could obtain employment ? Freedom to attend Roman Catholic religious services essential.—*Marie.*

You might apply to the Metropolitan Asylums Board, Victoria Embankment, E.C., or to the clerk to the Asylums Committee, London County Council, 21 Whitehall Place, S.W.

*Signs and Terms.*

(226) I should be glad to know of an elementary book which will make me acquainted with the "signs and terms commonly used in prescriptions." I am preparing for the probationer's preliminary examination at St. Bartholomew's.—*C. V.*

Miss Honnor Morten's text-book, "The Nurse's Dictionary of Medical Terms and Nursing Treatment," would probably suit you. Price 2s., from the Scientific Press.

*Home for the Aged.*

(227) Can you recommend a Home for the Aged where an old lady of 82 could be looked after and taken care of ? Payment of about 15s. a week would be made.—*X. Y. Z.*

Perhaps some of our readers may be able to give information.

### Standard Books of Reference.

"The Nursing Profession: How and Where to Train." 2s. net; post free, 2s. 4d.
"The Nurses' Dictionary of Medical Terms." 2s.
"Burdett's Series of Nursing Text-Books." 1s. each.
"A Handbook for Nurses." (Illustrated.) 5s.
"Nursing: Its Theory and Practice." New Edition. 3s. 6d.
"Helps in Sickness and to Health." Fifteenth Thousand.
"The Physiological Feeding of Infants." 1s.
"The Physiological Nursery Chart." 1s.; post free, 1s. 3d.
"Hospital Expenditure: The Commissariat." 2s. 6d.
All these are published by THE SCIENTIFIC PRESS, Ltd., and may be obtained through any bookseller or direct from the publishers, 28 and 29 Southampton Street, London, W.C.

### Travel Notes.

By Our Travelling Correspondent.

### LXVII.—HOLLAND.—*Continued.*

### The First Day.

I am supposing that you have only a week at your disposal; therefore it is necessary to make a plan, so that nothing essential shall be left undone. If you have a fortnight's holiday, so much the better; you will not find time hang heavy on your hands.

Rotterdam is unfortunately a good deal spoiled, from the artistic point of view, by the modern improvements which the thrifty and practical Dutch have instituted during the last fifty years; but there is still much to interest the lover of the picturesque.

One entire day will suffice to view the more important sights of Rotterdam. First there is the Church of St. Laurence, with a good organ on which there is a recital free every alternate Friday at 2.30. Inside there are some fine monuments to the stout old admirals of the seventeenth century. Near the church is the Stadhuis, a purely modern building devoid of interest. Not far off is Boyman's Museum, with a valuable collection of pictures—not so good as those of the Hague and Amsterdam, but well worthy of attention.

The Groote Market is very peculiar from its construction, being on a vaulting, once a canal; and it is a very busy sight on market day, which, if I remember rightly, is on a Thursday. Be sure to visit it at its busiest hour. Your landlord will tell you the best time. You will, perhaps, be glad to hear that very many Dutch hotel keepers speak more or less English.

In the market is a good statue of Erasmus, erected in 1622. He was born in the Wyde Kerkstraat, at No. 3. The house is marked by a Latin inscription and insignificant statue.

To go to the Zoological Gardens you pass out of the city by the Delft gate; it makes a nice short walk of half an hour. The most fashionable and agreeable promenade is along the Boompjes: it is a quay extending a mile by the side of the Maas, and is full of interest from the shipping of all nations here gathered together.

### Trams and Steamboats.

These are innumerable, and take one in all directions. The chief station is near to the Exchange. Steam trams go to Schiedam, Overschie and Hillegersberg, &c. Steamboats are frequent and quick, both for long and short journeys. Dordrecht, Briel, Gouda, and more distantly to Middelberg, Arnheim, &c. In summer a military band plays in the park, but I am doubtful if it begins so early as Easter.

### The Second Day.

I should devote this to the Hague. Arrived there by train, you can save fatigue by taking a tram into the town. One of the most interesting buildings is the Binnenhof. It has been a good deal restored of late. The original building was begun by Count William of Holland early in the thirteenth century. The memory of two gruesome tragedies clings to the old walls. In 1619 John van Oldenbarneveld was executed here. It is a long and sad story, admirably told by Mottey in a volume bearing the unfortunate minister's name. The aged victim was considerably over seventy, and was executed for the crime of "having conspired to dismember the States of the Netherlands." Fifty-three years later Cornelis de Witt was declared by the States-General sitting in the Binnenhof, to be guilty of traitorous conspiracy, and was immured in the Gevangenpoort, a tower near by. His brother, alarmed at the attitude of the States-General, hastened to the prison to see what steps could be taken to clear him, and the infuriated mob, breaking into the Gevangenpoort, literally tore the brothers in pieces. Their mangled remains were buried in the Nieuwe Kerk.

### The Famous Picture Gallery.

This is an interesting building called the Mauritshuis. I must not be beguiled into saying much of the contents. Rembrandt and Potter, Jan Steen, Gerard Don, and Van Ostade, are all well represented. Rembrandt's "School of Anatomy" is here, as well as the celebrated "Bull" of Paul Potter. This gallery is open free every day from 10 to 3.

### The Town Hall.

This was erected in 1565, and must therefore have witnessed many of the events of that stirring time. It is considered one of the best specimens of Dutch municipal architecture. The two side façades are very rich, and through the south entrance you will come into a small vestibule which contains an ancient bench belonging to the Sheriff's Court. Close by is the Fish Market, where all the fishwives of Scheveningen congregate and keep up a shrill conversation over their wares. The Groote Kerk is not specially interesting—at least not to those who have studied the magnificent Gothic churches of France and Germany. The carved wooden pulpit is very interesting, and the frames of the many coats of arms belonging to the Knights of the Golden Fleece. I should not waste time over the museum unless you have more than a week in Holland, and the same may be said of the Zoological and Botanical Garden. If possible try to walk along the charming Haagsche Bosch: it is only $1\frac{1}{4}$ miles long. Sunday is a good day because of the band. If you can pay two visits to the Hague you will perhaps have time for a very pleasant excursion by steam tramway to Scheveningen.

### TRAVEL NOTES AND QUERIES.

Rules in Regard to Correspondence for this Section.—All questioners must use a pseudonym for publication, but the communication must also bear the writer's own name and address as well, which will be regarded as confidential. All such communications to be addressed "Travel Editor, 'Nursing Mirror,' 28 Southampton Street, Strand." No charge will be made for inserting and answering questions in the inquiry column, and all will be answered in rotation as space permits. If an answer by letter is required, a stamped and addressed envelope must be enclosed, together with 2s. 6d., which fee will be devoted to the objects of the "'Hospital' Convalescent Fund." Any inquiries reaching the office after Monday cannot be answered in "The Mirror" of the current week."

Dinan for the Winter (Fighting Fifth).—If you can make suitable terms at the Hôtel de la Poste you could not do better. I have been there several times, and always found it comfortable, and the proprietor most obliging. I cannot say without knowing more of your needs, but I should think 6 francs a day would be about just.

Grindelwald in May (Yungfrau).—June is better than May, because the flowers are at their best in that month. It is rather a short time in which to go so far—three days each way from Scotland, or two and a half, if you travel by night from the North. The cheapest return fare from London is £5 12s. 6d. I will make enquiries for you about early arranged tours, so watch this column, and when they are published I will put in a notice for you; it will be in about three weeks. The flowers in Switzerland in June are a dream of beauty. Thanks for your kind remarks, it is always a great pleasure to hear one has been the cause of it to others.

Abroad for Six Months (Doubtful).—It cannot be done in Italy or Germany under £50, exclusive of your travelling expenses. This sum allows you, roughly speaking, $7\frac{1}{2}$ francs or lire per day, and though in some places you may manage for less, in others it will cost you more, to say nothing of incidental expenses. You must not think of embarking on such an undertaking with less. Such towns as Bologna or Orviete in Italy, and many places in the Black Forest or the Tyrol would suit you. In France, where, as I gather, you understand the language, I should recommend South Brittany or some quiet villages in the Loire Valley. Let me hear further.

Spain for Ladies Alone (Enterprise).—I do not think it is at all true that travelling alone in Spain is disagreeable. I travelled in very secluded parts with three other ladies, two of them quite elderly and by no means active, and we never experienced any annoyance. The Spaniards are most courteous and dignified. We greatly dared to travel third class, too, though warned that it would be intolerable. One thing, let me advise you, learn a little, even if but very little, Spanish before you start, just so as to be able to ask for tickets at a station and to choose your rooms in hotels. Only in Madrid and Seville, and not always there, is any French understood. The Spaniard is a non-progressive person. Warm clothes are very essential. North Spain is bitterly bleak and cold.

